# *Tetracha* Hope 1838 of the Turks and Caicos Islands (Coleoptera, Carabidae, Cicindelinae)

**DOI:** 10.3897/zookeys.147.2104

**Published:** 2011-11-16

**Authors:** Robert D. Ward1, Robert L. Davidson, David Brzoska

**Affiliations:** 1 Research Associate, Section of Invertebrate Zoology, Carnegie Museum of Natural History; Home address: 41708 North Signal Hill Court, Anthem, AZ 85086 USA; 2 Section of Invertebrate Zoology, Carnegie Museum of Natural History, 4400 Forbes Avenue, Pittsburgh PA 15213 USA; 3 Research Associate: Carnegie Museum of Natural History; Florida State Collection of Arthropods; University of Kansas Biodiversity Institute, Division of Entomology; Home address: 2740 Island Pond Lane, Naples FL 34119 USA

**Keywords:** new species, new subspecies, *Tetracha*, *Neotetracha*, Turks and Caicos Islands

## Abstract

A new species, *Tetracha (Neotetracha) naviauxi*, and a new subspecies, *Tetracha (Tetracha) sobrina caicosensis*, are described from the Turks and Caicos Islands. The key to *Tetracha* species in Naviaux (2007) is adapted to accommodate *Tetracha naviauxi*. *Tetracha sobrina caicosensis* is compared to other Caribbean subspecies of *Tetracha sobrina*.

## Introduction

In 1976, while on a pre-doctoral fellowship at the Smithsonian Institution, the senior author was studying New World megacephalines when he came across a single specimen from the Turks and Caicos Islands that did not fit any known species. The somewhat damaged singleton was collected on the Van Vost-American Museum of Natural History Bahama Islands Expedition (Rabb and Hayden 1957). Reluctant to describe it from a single specimen, Ward set it aside in hopes of collecting more material, or at least finding more specimens in other collections. Years later, in the early era of his long association as a Research Associate of Carnegie Museum of Natural History, he showed the specimen to Davidson, and there was another flurry of wishful thinking about travelling to this out-of-the-way locality specifically to search for this beetle. Such an expedition was never possible, and no further specimens ever turned up in other collections. Fortunately, in 2009, Dave Brzoska, also a Research Associate at Carnegie Museum, visited the museum and discussed this problem with Davidson. If the exact date and locality were given to him, he would commit the necessary time and expense to travel to this relatively out-of-the-way locality to search for this elusive beetle. In January of 2010, he did so, with great success as will be seen below. Serendipitously, along with the new species, Brzoska collected a good series of *Tetracha sobrina* from these islands, which we believe shows enough differences from other island populations to warrant a subspecific name. We take this opportunity in honoring Ross and Joyce Bell with a Festschrift to get the new species and subspecies described at long last, after a 35 year gestation period.

## Methods and terminology

Apparent body length was measured from the apex of the labrum to the apex of one elytron. We use here the actual sternum number, the first one visible being sternum II, and thus sterna II-VII can be seen ventrally (be careful in comparing this with papers using a simple numbering of the visible sterna, 1 being the first visible sternum (=Sternum II), and thus visible sterna 1–6). Mandibular teeth in *Tetracha* consist of an apical tooth, two or three terebral (incisor) teeth (see Acorn and Ball 1991 for a general discussion), a premolar and a complex molar. Premolars and molars are not further discussed here. The terebral teeth lie along the dorsal edge of the mandibular blade and are described here. Naviaux (2007) uses the term “*dent interne*” to describe terebral teeth.

The following acronyms are used:

AMNH Department of Entomology, American Museum of Natural History, Central Park West at 79th Street, New York, New York 10024, USA.


CMNH Section of Invertebrate Zoology, Carnegie Museum of Natural History, 4400 Forbes Avenue, Pittsburgh, Pennsylvania 15213, USA.


DBC Dave Brzoska Collection, 2740 Island Pond Lane, Naples, Florida 34119, USA.

FSCA Department of Entomology, Florida State Collection of Arthropods, FDACS-DPI, 1911 S. W. 34th St., Gainesville FL 32614, USA.


RNC Roger Naviaux Collection, 73, rue M. Dormoy, 03410 Domérat, France.


SEMC Division of Entomology, Public Safety Building, 1501 Crestline Drive, Suite 140, Kansas University, Lawrence, Kansas 66045, USA.


### Collecting History

Sometimes the places at which a species was not found can prove as important in the eventual big picture as the places at which it was found, so with this in mind we provide here a brief history of the collecting efforts for *Tetracha naviauxi*. The first and for many years only specimen was collected on February 11, 1953, a male taken by the American Museum of Natural History Van Vost Expedition . It was found on South Caicos Island, and there are no details regarding habitat. Over fifty years later, Dave Brzoska flew via Providenciales to South Caicos Island, where from January 5–8, 2010, he searched both at night and during the day for tiger beetles. He collected around dirt roads and salt flats near Victoria Salina, but in spite of the fact that it was only a few weeks earlier than the one known date for the species, he was unable to find any *Tetracha naviauxi*. He did, however, find 32 individuals of a new subspecies of *Tetracha sobrina* active at night, as well as some *Cicindela boops* and *Cicindela trifasciata* both at night and more commonly during the day. He then travelled to Salt Cay, where from January 9–11 he continued his search. Here he had much better luck and took 38 individuals of *Tetracha naviauxi* at three different sites, as elaborated below under the species description, again along with *Cicindela boops* and *Cicindela trifasciata.* He moved on from January 12–14 to Grand Turk Island, where again no *Tetracha naviauxi* could be found, but he did turn up another 13 individuals of the new subspecies of *Tetracha sobrina*. Here no *Cicindela boops* were found, and only a single specimen of *Cicindela trifasciata.* Last, he checked out two more islands (connected by a bridge) from January 15–18, North Caicos Island and Middle Caicos Island. No *Tetracha* at all were found on these islands, with *Cicindela trifasciata* the only tiger beetle seen.

## Taxonomy

### Tetracha (Neotetracha) naviauxi sp. n.

urn:lsid:zoobank.org:act:E689575B-F737-4BA7-AFC6-B4DEA7C4C281

http://species-id.net/wiki/Tetracha_naviauxi

**Figures 1–2.**
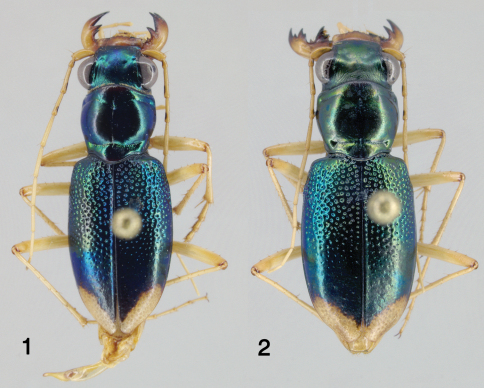
Dorsal habitus of *Tetracha naviauxi*; male, length 13.5 mm **1** female, length 14.1 mm **2**

Figs 1, 2 4 [Fig F4] –8

#### Type Material.

**(38 specimens)** Holotype ♂: “TURKS & CAICOS/ ISLANDS, Salt Cay-/ N. Creek Salina/ 21°19.6'N, 71°12.2'W/ D. Brzoska 9-I-2010” (CMNH). Allotype ♀: same data as holotype (SEMC). Paratypes (36) as follows: 20 same data as holotype (9 ♂♂, 4 ♀♀ DBC; 1 ♂, 1 ♀ CMNH; 2 ♂♂ FSCA; 2 ♂♂ RNC; 1 ♂ SEMC); 10 labelled: “TURKS & CAICOS/ ISLANDS, Salt Cay-/ Pilchard Hole Salina, 1m/ 21°18.7'N, 71°12.8’W/ D. Brzoska 9-I-2010” (7 ♂♂ DBC; 2 ♂♂ AMNH; 1 ♂ CMNH); 6 labelled: “TURKS & CAICOS/ ISLANDS, Salt Cay-/ near airport/ 21°20.0'N, 71°12.3'W/ D. Brzoska 9-I-2010” (4 ♂♂, 1 ♀ DBC; 1 ♂ CMNH).

#### Additional material.

**(1 specimen)** “Turks & Caicos Isls./ South Caicos Isl./ February 11, 1953/ / Van Vost--A. M. N. H./ Bahama Isls. Exped./ Coll. E. B. Hayden” (1 ♂ AMNH).

#### Type locality.

Salt Cay, Turks & Caicos Islands group.

#### Distribution.

Known only from two islands in the Turks & Caicos Islands group: Salt Cay and South Caicos Island.

#### Etymology.

A Latinized eponym, genitive case, based on the surname of Roger Naviaux.

#### Diagnosis.

**Figure 3.**
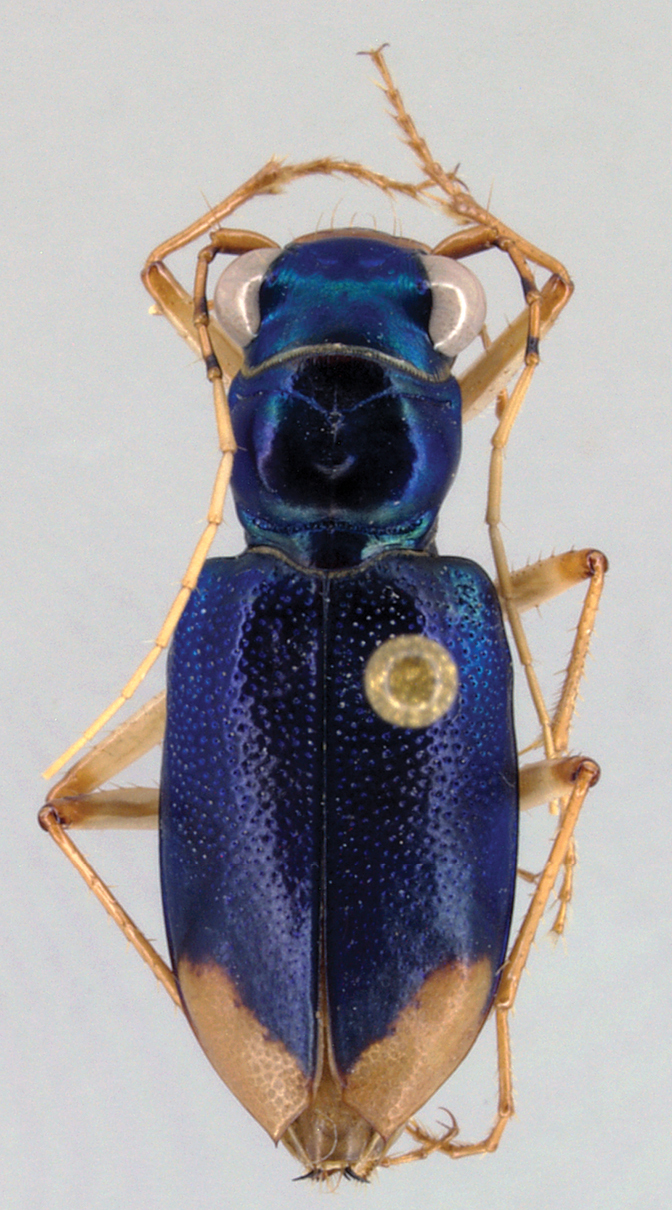
Dorsal habitus of *Tetracha acutipennis* female, length 14.8 mm

This species is easily distinguished from other Caribbean *Tetracha* by the relatively broad and squat elytra (Figs 1–2) with somewhat arcuate sides (elytra slenderer and longer with sides parallel in other species); the very weakly projected humeral angle; the reduced metathoracic wings (less than the length of an elytron, without reflexed apex); and the narrowly rounded, subacute elytral apex, whose apical extremity is some distance from the suture (similar only in *Tetracha acutipennis* (Dejean)(Fig. 3) and *Tetracha misella* Naviaux, in which the elytral apex is drawn out into a spine or sharp point; other species have broadly rounded apices curving shallowly into the suture).

#### Description.

(Figs 1–2). Length (n=15) males 11.9–14.6 mm; females (n=7) 13.4–14.6 mm. Small-sized, robust *Neotetracha*. Head and pronotum dorsally metallic blue-green, anterior margin of pronotal collar black to ferruginous; elytra bright metallic blue to blue-green for most of length, blackish-green between apical lunules; ventral surface metallic green on head, prothorax and laterally on sterna II-IV; center of sterna III-IV, all of sternum V, most of sternum VI, and medial base of sternum VII brown; posteriolateral corner of sternum VI and broad band along posterior margin of sternum VII pale; antennae, palpi, mandibles, labrum and legs mostly pale (coxae brunneus). Head, thorax and abdomen glabrous except for usual fixed setae, fringes of cleaning setae along anterior and posterior margins of prothorax, and elytra with a subsutural row of minute setigerous punctures and a few basal and humeral setae.

**Figures 4–5.**
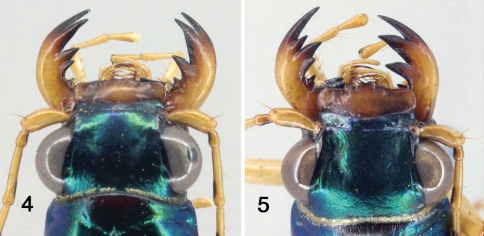
Mandibles of *Tetracha naviauxi*, male **4** female **5**

Head. Left mandible of male (Fig. 4) with four teeth, apical tooth and first terebral tooth elongate, subequal in length, apical tooth deflexed ventrally, terebral teeth of decreasing size, third terebral tooth basally anastomosed to second; right mandible of male (Fig. 4) with elongate apical tooth and two broad incisor-like terebral teeth, apical and first terebral tooth deflexed ventrally. Left mandible of female (Fig. 5) with apical tooth elongate, slightly deflexed ventrally, first and third terebral teeth shorter than second, third terebral tooth anastomosed to second; right mandible (Fig. 5) similar to that of male. Basal molars of each mandible with tuft of setae at base. Mandibular scrobes shallow, without setae or setal pores, dorsal edge rounded nearly to base. Labrum with four submarginal setae; labrum short, sides subparallel, anteriolateral angles rounded, posterior margin shallowly V-shaped; labrum of male narrow with four small subequal teeth; female with teeth more sharply defined, medial pair anteriorly produced. Clypeus narrow, chevron-shaped, laterally depressed. Tentorial pits deep, fronto-clypeal suture effaced between pits, evident laterally, bisetose; two supraorbital setae over each eye connected by shallow groove paralleling the eye; frontal suture weakly indicated by pair of elongate depressions. Frons and vertex mostly smooth, vertex sculptured with web of very shallow irregular “cracks.” Microsculpture of frons and vertex irregularly isodiametric (weakest near eyes). Eyes large, convex. Antennae entirely testaceous except for dark spots toward apices of antennomeres 2–4; antennomeres 1–4 without dorsal carina. Mentum with median lobe acute, lateral lobes not strongly acuminate; ligula bisetose.

Thorax. Pronotum strongly convex, cordiform; anterior transverse impression weak medially; median longitudinal impression shallow, extended to anterior margin; posterior transverse impression deep with triangular lateral basal impressions at the posteriolateral corners; anterior collar with shallow impressed transverse submarginal line extended to anteriolateral corner but effaced medially; posterior collar with marginal line effaced medially. Notopleural suture deep; anterior corner of proepisternum separates pronotum from prosternum; proepisternum with a few large shallow punctures in posterioventral corner. Coupling sulcus of female a shallow groove along mesopleural suture from mesocoxa to humerus, with trace of small pit under humerus.

Legs. Pro- and mesotrochanters with small subapical setigerous punctures. Males with protarsomeres 1–3 with full tarsal pads, protarsomeres 2–3 asymmetrical.

Elytra. Elytra relatively short and broad compared to combined length of head and thorax, gently curved laterally from humerus to apex, widest at middle; humerus rounded and weakly projected, less protruded than in related species; apex obtusely angulate with angle evenly rounded, not acuminate, oblique to terminus of sutural line, apical extremity quite some distance from suture; apex without microserrulation. Apical lunules narrow, parallel-sided, attaining elytral suture. Anterior half of elytron with well spaced, large, deep pits (distance between pits 1–3 times diameter of average pit), each with a small reflective spot and minute irregularity at the bottom, pits irregular without tendency to form rows; posterior half with smaller punctures rapidly decreasing in size and becoming impunctate proximal to apical lunules; elytral surface flat and smooth between punctures, lacking imbrications, surface somewhat shiny.

Metathoracic Wings. Wings reduced to a narrow elongate pad about 3/4 length of an elytron.

Abdomen. Sterna IV-VI each with pair of ambulatory setae. Sternum VII broadly and deeply notched medially in male, small shallow medial emargination in female.

**Figures 6–7. F4:**
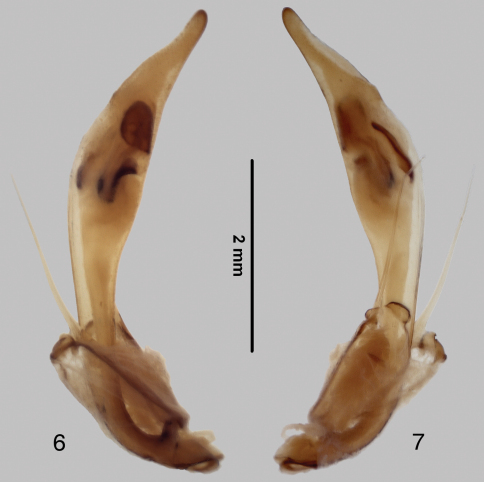
Male genitalia of *Tetracha naviauxi*, left lateral view **6** right lateral view **7**

Aedeagus. (Figs 6–7). Elongate and slender, broadest at middle, apically drawn out to blunt, rounded tip; apex long and narrow, slightly deflexed.

#### Variation.

The single specimen from South Caicos Island differs in a few minor details from the series from South Cay, and we have therefore left it out of the type series. Whether these details have any significance will have to wait for a more robust sample and a better understanding of which islands are inhabited by the species. Though there is some variation, all the specimens from South Cay are a greenish-blue, whereas the South Caicos individual is a bright dark blue. The border between the dark elytron and its apical lunule is slightly jagged and diffuse in the South Cay specimens, whereas the border is much smoother and more sharply defined in the South Caicos individual.

#### Dedication.

We take great pleasure in naming this remarkable and beautiful new species after our friend and colleague Roger Naviaux in recognition of his many years of fine work on tiger beetles, and in particular in recognition of his excellent monograph of *Tetracha* (Naviaux 2007).

#### Discussion.

*Tetracha naviauxi* fits into the *Tetracha acutipennis* group as defined by Naviaux (2007: 80). Characters are similar in the conformation of the mandibles, the shape of the labrum, the dorsal coloration (green foveae), the shape of the apical lunule, the deep non-granular punctures with reflective spot at the base of each and smooth surface around each puncture, the third protarsomere slightly asymmetrical, the long slender shape of aedeagus, and the internal sclerites of the aedeagus. Most important, the structure of the elytral apex is similar: the elytral apex is narrowly rounded at some distance from the suture, and thence curved forward to the suture leaving a relatively broad incision between the two elytra. *Tetracha naviauxi* (Figs 1–2) differs from the other two species in this group (*Tetracha acutipennis* (Fig. 3) and *Tetracha misella*) most obviously by its gestalt. The latter two species have longer, slenderer, parallel-sided elytra with the apex drawn out into a spine or at least an acute angle, whereas *Tetracha naviauxi* has shorter, broader elytra with the sides gently arced from humerus to apex, and the apex is at most subacute without a spine. All specimens of *Tetracha naviauxi* have reduced metathoracic wings, narrow and shorter than the elytron, without reflexed apex, with the accompanying reduction of the length of the metepisternum and a weak, barely projected humerus; in *Tetracha acutipennis* and *Tetracha misella*, all known specimens are fully winged, with a slightly longer metepisternum and a much stronger, more projected humerus. *Tetracha naviauxi* has sparser, deeper, larger elytral punctures, which disappear relatively abruptly in the apical third; elytral punctures in the other two species are denser, shallower, smaller, and reduced in size in the apical third but present nearly to the apex. The coupling sulcus of female *Tetracha naviauxi* is shallower, less abruptly angled against the mesepisternum, and ends laterally under the humerus in only a trace of a small pit; female *Tetracha acutipennis* have a deeper and more sharply angled sulcus ended laterally under the humerus in a pronounced pit (we have not seen the sulcus for *Tetracha misella*, but presumably it is similar to *Tetracha acutipennis* as Naviaux does not mention any difference). The aedeagi of *Tetracha naviauxi* and *Tetracha acutipennis* are similarly shaped, with internal sclerites in the same positions and only minor differences in shape. The external aedeagal tube of *Tetracha acutipennis* is, however, considerably more elongate than that of *Tetracha naviauxi*, especially both basally and apically compared with the swollen central bulb; this is most easily seen at the apex, where *Tetracha acutipennis* tapers to a longer and slenderer point, whereas *Tetracha naviauxi* is shorter, thicker, and with the tip slightly thickened and curved.

This is so far as we know the only short-winged tiger beetle known from the Caribbean region. The structure of the humeral region, the metathoracic plates and the elytra suggests that the species is always short-winged, and that it is unlikely that fully-winged individuals might occur from time to time. Loss of flight has certainly curtailed its ability to disperse and in part accounts for its limited distribution on these remote islands.

We include here a modified version of Naviaux’s (2007: 80) key to species groups of *Neotetracha* to accommodate the new species.

**Table:** 

2	Mandibles of male not or moderately forked; elytral apex rounded into suture at an obtuse angle	5
–	Mandibles of male deeply and broadly forked, the first terebral tooth approximately of same length as the apical tooth; elytral apex narrowly rounded and subacute or spined distant from the suture OR broadly rounded into suture at a right angle	6
6	Elytral apex narrowly rounded and subacute or acuminate, apical extremity distant from suture	*acutipennis* group 6’
–	Elytral apex broadly rounded into suture at a right angle, apical extremity much closer to suture	13
6’	Elytral apex narrowly rounded or subacute, but without sharp angle or acuminate spine; elytra broader, shorter, with sides gently curved from humerus to apex; humerus less protruded; elytra punctation coarser, sparser, deeper, weak or absent in apical third; metathoracic wing reduced; Turks and Caicos Islands	*Tetracha naviauxi* new species
–	Elytral apex forming an acute angle or acuminate spine; elytra slenderer, longer, with sides parallel; humerus more strongly protruded; elytra punctation smaller, denser, shallower, weaker but still obvious and present over most of apical third; Greater Antilles	6’’
6’’	Elytral apex less rounded, drawn into a long point; length 13–15.5 mm; Hispaniola and Cuba	*Tetracha acutipennis* (Dejean)
–	Elytral apex more rounded, drawn into a short point; length 10.5–12.6 mm; Haiti	*Tetracha misella* Naviaux

#### Habitat and collecting notes.

**Figure 8.**
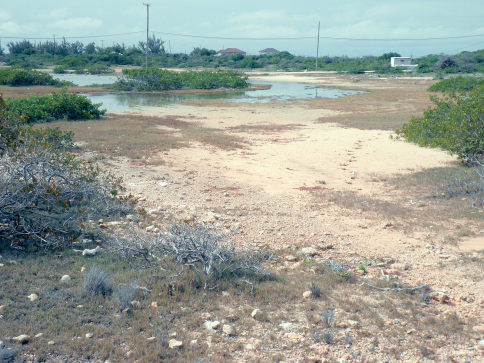
Habitat of *Tetracha naviauxi*, Salt Cay, airport

At North Creek Salina on Salt Cay, taken mostly on moist salt flat covered with a dark algal mat surrounded by *Salicornia*. At Pilchard Hole Salinas, taken on damp salt flats. At the airport site (Fig. 8), taken at a small salt pond and adjacent flats. All three sites also had *Cicindela boops* and *Cicindela trifasciata*. The latter were taken mostly during the day with a few at night, but all *Cicindela naviauxi* were taken at night.

### Tetracha (Tetracha) sobrina caicosensis subsp. n.

urn:lsid:zoobank.org:act:FE4266B8-95D4-4428-B30F-A4E26FA55B52

http://species-id.net/wiki/Tetracha_sobrina_caicosensis

Figs 9 –11

#### Type material.

**(45 specimens)** Holotype ♂: “TURKS & CAICOS/ ISLANDS, South Caicos-/ Victoria Salina, 1m/ 21°29.6'N, 71°31.5 W/ D. Brzoska 5-7-I-2010” (CMNH). Allotype ♀: same data as holotype (SEMC). Paratypes (43) as follows: 30 same data as holotype (12 ♂♂, 7 ♀♀ DBC; 2 ♂♂, 2 ♀♀ CMNH; 1 ♂, 1 ♀ AMNH; 1 ♂, 1 ♀ FSCA; 1 ♂, 1 ♀ RNC; 1 ♂ SEMC); 13 labelled: “TURKS & CAICOS/ ISLANDS, Grand Turk-N./ end Saunders Pond, 1m/ 21°29.1'N, 71°08.9'W/ D. Brzoska 11-13-I-2010” (11♂♂, 1♀ DBC; 1 ♂ CMNH).

#### Type locality.

South Caicos Island in the Turks & Caicos Islands group.

#### Distribution.

Known only from two islands in the Turks & Caicos Islands group: South Caicos Island and Grand Turk Island.

#### Etymology.

A geographic name formed from “Caicos,” the islands on which this subspecies has been found, and the Latin suffix “-*ensis*,” meaning of or from that place.

#### Diagnosis.

This new subspecies is similar to the other six subspecific taxa of *Tetracha sobrina*, closest to *Tetracha sobrina infuscata* (Mannerheim), but differs in the reduced size of its apical lunules (narrow, more or less parallel-sided), green (not blue) elytral margins; small, round, basal trans-sutural coppery-red spot; and broad, black medial band.

#### Description.

**Figures 9–10.**
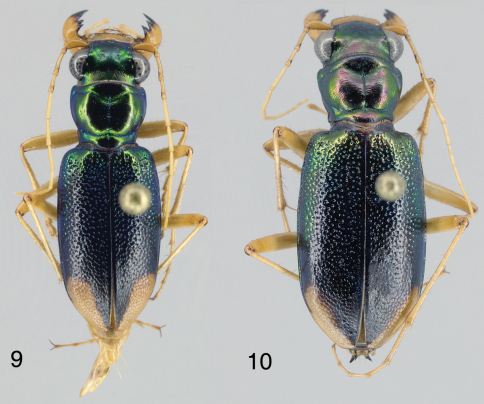
Dorsal habitus of *Tetracha sobrina caicosensis*; male, length 14.5 mm **9** female, length 16.0 mm **10**.

(Figs 9–10). Length males (n=15) 13.3–15.8 mm; females (n=13) 14.1–16.4 mm. Most similar to *Tetracha (Tetracha) sobrina infuscata* (Mannerheim). All appendages pale except antennomeres 2–4 which have small distal brown spots opposite eyes and distal tips of femora which are infuscated. Clypeus and frons metallic green; vertex coppery, duller (South Caicos) to redder (Grand Turk), but never the bright red of *Tetracha sobrina infuscata*. Pronotum red with green highlights, especially the posterior transverse line, but again not the bright red of *Tetracha sobrina infuscata*. Elytra (excluding green punctures) with base with a small trans-sutural, rounded red spot; lateral margins, between the elytral base and the anterior tip of the apical lunule, clearly metallic green; the remainder of the elytra black, no violet areas toward the elytral apices; apical lunule narrow, parallel-sided, inner margin less distinct with scattered disjunct pale cells. Elytral sculpturing consists of punctation over basal half, pits deep blue-green; pits separated by smooth, shiny metallic surface, with very little trace of imbrications or granules; punctures are weakly aligned transversely; posteriorly, pits decrease in size and density while becoming increasingly imbricated, and merge into shingle-like transverse rows, especially between the apical lunules; pits and imbrications greatly reduced to absent on lunules.

#### Discussion.

The characters separating this subspecies are somewhat superficial ones of color and texture, but as this is largely all that separates the six described subspecies of this wide-ranging species (see Naviaux 2007), and as these characters seem to be relatively constant over large populations, it seems appropriate enough to describe these isolated island populations from the Turks and Caicos Islands as a new subspecies. The new subspecies is easily separated from five of the six currently recognized subspecies (Naviaux 2007) by the very black color of the elytra, starting in front of the apical lunules and reaching more or less to the base along the suture, and by the relatively narrow and parallel-sided apical lunules. The other five subspecies all have bright metallic colors along the elytral suture, and broader, rounded apical lunules, in most individuals more comma-shaped or globular (these are three mainland subspecies (*Tetracha sobrina punctata* (Laporte), *Tetracha sobrina freyi* (Mandl) and *Tetracha sobrina guyanensis* Naviaux), one subspecies both mainland and Lesser Antilles (*Tetracha sobrina sobrina* (Dejean)), and one subspecies in the Lesser Antilles (*Tetracha sobrina antiguana* Leng and Mutchler)). The most similar subspecies is *Tetracha sobrina infuscata* (Mannerheim) from Cuba, Hispaniola, Puerto Rico and the Virgin Islands. Our new subspecies differs from this mainly in the extent of the blackened center of the elytra, and in the shape of the apical lunules. *Tetracha sobrina caicosensis* has the elytra black from the apical lunules to at least the anterior third, and in many individuals more or less all the way to the base, and the green marginal band is very narrow. The apical lunules are narrow and usually parallel to the elytral margin; in some individuals somewhat broader, but still always narrower than in *Tetracha sobrina infuscata*, with the incision between the two lunules wider and the medial border much more jagged or irregular. *Tetracha sobrina infuscata* has the elytra blackened just in front of the apical lunules, but only in the apical third or so, becoming gradually more metallic green in the anterior two-thirds, and the green marginal band is broader. The apical lunules are very wide, with a relatively narrow incision between the two lunules, and with the medial border very sharply defined and regular.

#### Habitat and collecting notes.

**Figure 11.**
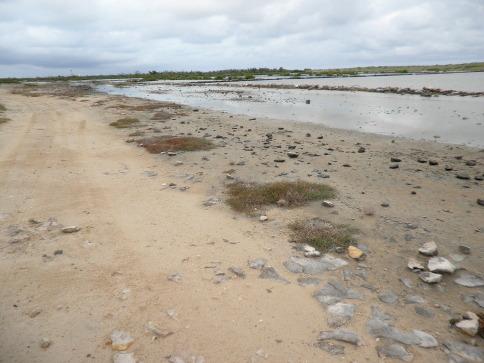
Habitat of *Tetracha sobrina caicosensis*, Grand Turk

At Victoria Salina on South Caicos Island, taken on the edges of dirt roads, mostly at a large open dry sandy area away from the wet salt flats. *Cicindela boops* and *Cicindela trifasciata* were taken nearby, but in moist saline areas*.* The *Cicindela* were taken mostly during the day, with a few at night; the *Tetracha* were all taken at night. At Saunders Pond on Grand Turk Island (Fig. 11), taken at the edge of the pond and at damp saline areas, some with green algal mats. This is a much wetter habitat than Victoria Salina. A single *Cicindela trifasciata* was also taken.

## Supplementary Material

XML Treatment for Tetracha (Neotetracha) naviauxi

XML Treatment for Tetracha (Tetracha) sobrina caicosensis
